# Hyponatremia and intensive care outcome

**DOI:** 10.1186/cc12381

**Published:** 2013-03-19

**Authors:** P Dean, R Docking, D Govenden, A Davidson, A Mackay

**Affiliations:** 1 Victoria Infirmary, Glasgow, UK

## Introduction

Disorders of sodium (Na^+^) and water homeostasis are common in hospitalised patients. Hyponatremia in particular has been associated with worse hospital outcome and length of stay [1]. We aimed to define the incidence of hyponatremia (serum Na^+ ^≤134 mmol/l) in our intensive care population and to determine whether it was associated with ICU outcome or length of stay.

## Methods

Demographics, APACHE II score, outcome data and admission sodium were retrieved from the Ward Watcher system in the Victoria Infirmary ICU for 2,440 consecutive admissions from January 2005 to present. We divided patients into three groups depending on serum Na^+ ^(≤134 mmol/l, 135 to 144 mmol/l, ≥145 mmol/l) and compared APACHE II score, length of stay and ICU outcome between patients with a low versus a normal serum Na^+^. Data were analysed using the chi-squared test, Student's *t *test and the Mann-Whitney test where appropriate.

## Results

Of the 2,440 patients studied, 1,993 had APACHE II data and serum Na^+ ^recorded and so were included for analysis. In total, 453 patients (22.7%) had a serum Na^+ ^≤134 mmol/l and 1,388 patients (67.1%) had a serum Na^+ ^of 135 to 144 mmol/l. Patients with a low Na^+ ^had a higher mortality (OR = 1.48, 95% CI = 1.16 to 1.90, *P *0.001), a higher APACHE II score (22 vs. 19, *P *0.001) and higher mean age (60 years vs. 58 years, *P *0.001) than patients with a normal serum Na^+^. Mean length of stay of patients with low serum Na^+ ^was also longer (5.1 days vs. 4.6 days) although this was not statistically significant (*P *= 0.09).

## Conclusion

In summary, hyponatremia is a useful index of severity of illness in our ICU population. Whether this is a direct adverse effect of low serum sodium levels, or if hyponatremia is simply a marker for 'sicker' patients, is not known.
